# *Rickettsia helvetica* and *R. monacensis* infections in immature *Ixodes ricinus* ticks derived from sylvatic passerine birds in west-central Poland

**DOI:** 10.1007/s00436-016-5110-6

**Published:** 2016-05-11

**Authors:** Beata Biernat, Joanna Stańczak, Jerzy Michalik, Bożena Sikora, Stella Cieniuch

**Affiliations:** 1 Department of Tropical Parasitology, Institute of Maritime and Tropical Medicine, Medical University of Gdańsk, Powstania Styczniowego 9B str, 81-519 Gdynia, Poland; 2 Department of Animal Morphology, Faculty of Biology, Adam Mickiewicz University, 89 Umultowska str, 61-701 Poznań, Poland

**Keywords:** *Ixodes ricinus*, *Rickettsia helvetica*, *Rickettsia monacensis*, Spotted fever group rickettsiae, Birds, Ticks

## Abstract

The aim of this study was to assess the importance of forest passerine birds in spreading ixodid ticks infected with rickettsiae of spotted fever group (SFG) in sylvatic habitats in western Poland. In total, 834 immature *Ixodes ricinus* ticks were found on 64 birds of 11 species which were captured during the tick-questing season between May and September of 2006. Ground-foraging passerines hosted most of the ticks compared with arboreal species, and therefore, only the former group was included into a detailed analysis. Significant predominance of larvae over nymphs was observed (581 vs. 253, respectively). Blackbirds and song thrushes hosted 82 % (*n* = 681) of the ticks collected from all infested passerines. The overall prevalence range of SF rickettsiae (including *Rickettsia helvetica* and *Rickettsia monacensis*) in bird-derived ticks was 10.5–26.9 %, exceeding that in questing ticks, and in ticks feeding on rodents and deer reported earlier from the same study area. This high prevalence of infection in immature *I. ricinus* ticks feeding on passerine birds strongly implies that they are involved in the enzootic maintenance of spotted fever group rickettsiae in the tick vector populations occurring in sylvatic habitats.

## Introduction

Birds, especially passerine migratory species, could be involved in carriage of microbial pathogens as biological reservoirs, when microorganisms multiply in their body, mechanical carriers, and hosts of epidemiologically important ixodid ticks (Hubálek 2004; Foti et al. 2011; Falchi et al. 2012). In Europe, *Ixodes ricinus* ticks detached from their avian hosts were found to harbor tick-borne encephalitis virus (TBEV) (Waldenström et al. 2007; Kazarina et al. 2015), different genospecies of *Borrelia burgdorferi* sensu lato (Comstedt et al. 2006; Poupon et al. 2006; Michalik et al. 2008; Heylen et al. 2012), *Anaplasma phagocytophilum* (Paulauskas et al. 2009; Mǎrcuţan et al. 2014), spotted fever group *Rickettsia* spp. (Elfving et al. 2010), *Babesia* spp. (Franke et al. 2010b; Hasle et al. 2011; Žėkienė et al. 2011), and others (Hasle 2013; Lommano et al. 2014; Berthová et al. 2016). In Poland, the only studies on the role of birds in ecology of tick-borne diseases were focused so far on Lyme borreliosis and *A. phagocytophilum*, the agent of granulocytic anaplasmosis (Gryczyńska et al. 2004; Skoracki et al. 2006; Michalik et al. 2008). The results obtained validated the concept of some avian-associated genospecies within the *B. burgdorferi* s.l. complex and demonstrated that passerine birds, like tree pipit, dunnock, chaffinch, and thrush species (*Turdus merula* and *Turdus philomelos*), may support the circulation of *B. garinii* and *B. valaisiana* under natural conditions as a reservoir of spirochetes and carriers of infected ticks. On the other hand, the authors failed to detect *A. phagocytophilum* DNA in avian blood and in ticks collected from passerine birds.

In this study, we aimed to assess the prevalence of *Rickettsia* spp. in ticks removed from birds captured in the sylvatic areas of western Poland. This paper is a follow-up study to our previous investigation on the role of deer, rodents, and ticks feeding on them in the perpetuation of the spotted fever group rickettsia in the same sylvatic habitat (Stańczak et al. 2008; Biernat et al. 2016).

## Material and methods

### Study area and bird sampling

**Fig. 1:**
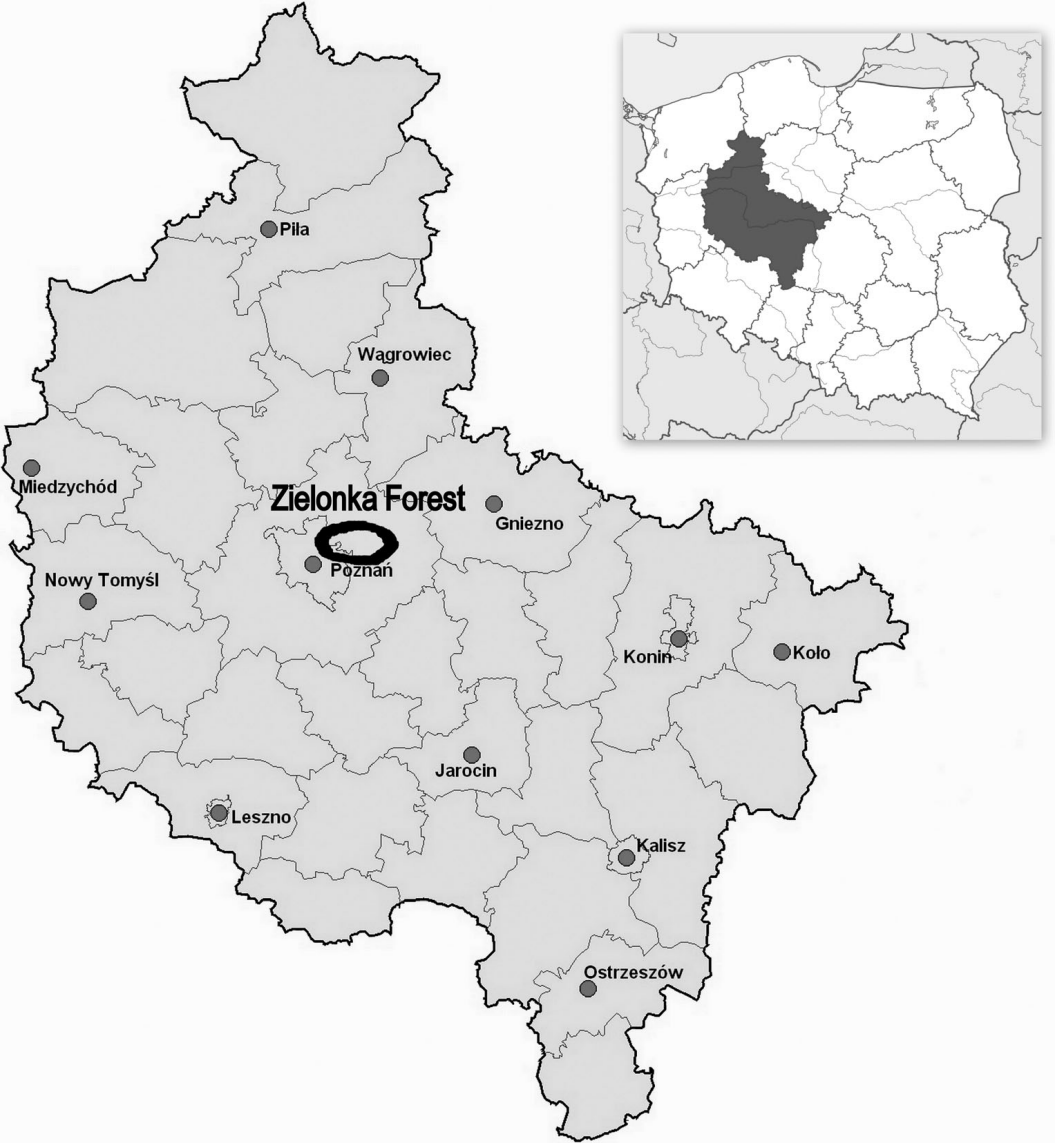
Location of the “Zielonka Forest”—the collection sites of birds, Wielkopolska province, west-central Poland

Birds were captured in two sylvatic sampling sites situated within the middle of 10,000-ha Landscape Park “Zielonka Forest”—a compact sylvatic area with predominance of mixed forests about 30 km away from the city of Poznań, Wielkopolska province, in west-central Poland (52° 17′ N; 16° 50′ E) (Fig. 1). Site 1 was chosen in a mixed forest stand (up to 50–60 years old) dominated by oak (*Quercus sessilis*) and Scots pine (*Pinus silvestris*), adjacent to a small settlement, Zielonka. Site 2 was the reserve “mixed forest in Łopuchówko Forest Division” which consists of a natural oak and pine forest stand (up to 200 years old) including also younger hornbeams (*Carpinus betulus*) and beeches (*Fagus sylvatica*).

Birds were sampled during their breeding season May–July, and in August and September of 2006 using 20 ornithological nylon mist-nets (each 12 × 3.5 m). Nets were placed along five transects (each 48-m long) spaced 15–60 m apart and were checked for netted birds at 1-h intervals from dawn until dusk. Each trapping session lasted 1 week. Birds were identified to species level and examined carefully for ticks, particularly around the eyes and bill where most ticks attach. Feeding specimens were detached with forceps and preserved in 75 % ethanol for further examination. A small amount of whole blood (approximately 10 μL) was collected from each bird via branchial vein puncture using a 28-gauge needle and a pipette. Blood samples were stored at −20 °C in Eppendorf tubes containing EDTA. Before release, the birds were marked by clipping an outer tail feather to indicate their recaptured status. Recaptured individuals during each mist-netting session were released immediately and not included in the infestation analysis. Trapping and handling procedures of birds were approved by the respective authorities (permission no. DOPog-4201-03-158/03/al). All collected blood samples had been examined for spotted fever *Rickettsia* DNA prior to present investigation (Stańczak et al. 2009).

### DNA extraction and real-time PCR amplification

Only undamaged ticks were tested by PCR. Each tick was examined under a stereomicroscope in order to check the level of engorgement. Based on their volume, ticks were subjectively determined as (i) unfed (without any visible blood remnants in the midgut), (ii) partially fed, and (iii) fully fed. Total DNA from fully engorged larvae and nymphs was isolated using Sherlock AX commercial kit (A&A Biotechnology, Gdynia, Poland). Extraction of nucleic acids from unfed and partially engorged specimens was made by boiling in NH_4_OH (Rijpkema et al. 1996). Altogether, 550 immature *I. ricinus* (293 larvae and 257 nymphs) were selected and tested for the presence of rickettsial DNA. Twenty-four larvae and 140 nymphs were examined individually. The remaining 386 ticks were tested in pools of two to five ticks (88 × 2; 43 × 3; 2 × 4; and 10 × 5, respectively). Larvae and nymphs as well as ticks from different birds were never mixed in the same pools.

All tick samples were screened by real-time PCR for the citrate synthase encoding gene (*gltA*) specific for all *Rickettsia* spp. Primers Rick GltA-f (5′-ATCCTACATGCCGATCATGAGC-3′) and Rick GltA-r (5′-GTGAGCAGGTCCCCAAAGTG-3′) were designed to target a 123-bp part of the gene with TaqMan probe (5-HEX-ATGCTTCTACTTCAACAGTCCGAATTGCCG-BHQ1-3′).

Reactions were performed in a total mixture volumes of 20 μL contained the Real-Time 2× PCR Mastermix Probe (A&A Biotechnology, Gdynia, Poland) 0.4 μL of each primer (10 μM), 0.2 μL of the probe (10 μM), 7 μL of water, and 2 μL of extracted DNA. Negative and positive controls were included in all runs. *Rickettsia*-positive control was constructed by cloning the 123-bp PCR amplicon into a circular pJet1.1 plasmids (Fermentas, USA) which were transformed into competent TOP10F’ *Escherichia coli* (Invitrogen). Then plasmids were extracted by using Plasmid Mini commercial kit (A&A Biotechnology, Gdynia, Poland). Concentrations of plasmids were measured with NanoDrop 1000 spectrophotometer (Thermo Scientific, USA). The control template was diluted 1:10. Reactions were performed in a Mx3005P Real-Time QPCR System (Stratagene, CA, USA). Cycling conditions included an initial activation of the *Taq* DNA polymerase at 95 °C for 10 min followed by 40 cycles of a 15 s denaturation at 95 °C followed by a 1-min annealing-extension step at 60 °C.

### Specification of *Rickettsia* spp.

Most real-time PCR-positive samples were next rerun using conventional or semi-nested PCR assays to obtain longer amplicons for further DNA sequencing. In some cases, however, we lacked templates for these assays. A conventional PCR was carried out using RpCS.877p and RpCS.1258n primers amplifying a 380-bp fragment of the *gltA* gene (Nilsson et al. 1999). Semi-nested PCR was conducted with three primers of which Ric and Ric U8 yielded a 1385-bp fragment encompassing almost the complete 16S rRNA gene, while Ric and Ric Rt flanked a 757-bp fragment. All PCR reactions were carried out in a GeneAmp^®^ PCR System 9700 (Applied Biosystems 850, Foster City, CA, USA) as previously described (Biernat et al. 2016). PCR products were separated on 2 % agarose gels stained with Midori green DNA Stain (Nippon Genetics Europe GmbH) and visualized under UV light using GelDoc–It, Imagine Systems UV^™^ Transluminator (Upland, CA, 91786, USA).

Not all positive samples were identified to species level by further DNA sequencing. The weak *Rickettsia*-positive amplicons were excluded from analysis. Chosen products were purified using the Clean-Up purification kit (A&A Biotechnology, Gdynia, Poland), sequenced in both directions with the same primers as in the PCR and semi-nested PCR assay by using ABI Prism^®^ Big Dye^™^ Terminator v.3.1 Cycle Sequencing Kit, and then analyzed with an ABI PRISM 3100 or 3130 *xL* genetic Analysers (Applied Biosystem^®^) according to the manufacturer’s protocol. Finally, sequences were edited and compared with each other and with corresponding sequences registered in the GenBank database using the NCBI BLAST program (US National Institutes of Health, Bethesda, Maryland) [http://blast.ncbi.nlm.nih.gov/Blast.cgi]. Then, consensus sequences were submitted to GenBank.

## Results

### Birds and tick loads

In total, 148 birds of 18 species were mist-netted and examined for ticks between May and September 2006. Of these, 126 (92 %) representing 16 species belonged to the order Passeriformes whereas two (8 %) represented the order Piciformes (Table 1). The most commonly caught hosts were blackbirds (*T. merula*) and chaffinches (*Fringilla coelebs*) followed by European robins (*Erithacus rubecula*) and song thrushes (*T. philomelos*), which together accounted for 50 % of all examined.

**Table 1:** Birds trapped in “Zielonka Forest”, west-central Poland, in 2006 and data on their infestation by *Ixodes ricinus* ticks

Birds			*Ixodes ricinus*		
			No. (%) infested birds/no. ticks/no. ticks per infested bird		
Family	Species—no. infested/examined birds		Larvae	Nymphs	Total
Turdidae^a^	Blackbird (*Turdus merula*)^c^	26/26	25 (96.2)/371/14.8	26 (100)/179/6.9	26 (100)/550/19.2
	Song thrush (*Turdus philomelos*)^c^	10/13	10 (77)/88/8.8	10 (77)/43/4.3	10 (77)/131/13.1
Sturnidae^a^	Starling (*Sturnus vulgaris*)^c^	6/6	4 (66.7)/11/2.8	6 (100)/15/2.5	6 (100)/26/4.3
Fringillidae^a^	Chaffinch (*Fringilla coelebs*)^c^	6/23	5 (21.7)/21/4.2	2 (8.7)/3/1.5	6 (26.1)/24/1.2
	Hawfinch (*Coccothraustes coccothraustes)*	3/5	3 (60)/11/3.7	2 (40)/2/1	3 (60)/13/4.3
Muscicapidae^a^	Robin (*Erithacus rubecula*)^c^	7/14	6 (42.)/35/5.8	5 (35.7)/5/1	7 (50)/40/5.7
Paridae^a^	Blue tit (*Parus caeruleus*)	1/2	1 (50)/9/9	0	1 (50)/9/9
	Coal tit *(Parus ater*)	0/1	0	0	0
Phylloscopidae^a^	Chiffchaff (*Phylloscopus collybita*)	0/1	0	0	0
Certhiidae^a^	Treecreeper (*Certhia familiaris*)	1/12	1 (8.3)/5/5	0	1 (8.3)/5/5
Emberizidae^a^	Yellowhammer (*Emberiza citrinella*)	0/6	0	0	0
Motacillidae^a^	Tree pipit (*Anthus trivialis*)^c^	1/1	1 (100)/31/31	1 (100)/5/5	1 (100)/36/36
Sylviidae^a^	Blackcap (*Sylvia atricapilla*)	1/6	1 (16.7)/2/2	0	1 (16.1)/2/2
	Whitethroat (*Sylvia communis*)	0/4	0	0	0
	Garden warbler (*Sylvia borin*)	0/2	0	0	0
Sittidae^a^	Wood nuthatch (*Sitta europaea*)	2/9	1 (50)/1/1	1 (50)/1/1	2 (22.2)/2/1
Picidae^b^	Great spotted woodpecker (*Dendrocopos major*)	0/10	0	0	0
	Black woodpecker (*Dryocopus martius*)	0/1	0	0	0
Total		64/142	58 (40.8)/585/10.1	53 (37.3)/253/4.7	64 (45.1)/838/12.8

^a^Order Passeriformes

^b^Order Piciformes

^c^Ground-foraging species

Altogether, 64 (43.2 %) birds representing 11 species had attached 834 ticks (Table 1). The infested birds were parasitized exclusively by immature *I. ricinus* (581 larvae and 253 nymphs). The larva/nymph ratio was 2.3, indicating significant predominance of larvae over nymphs. The tick burden was significantly higher (*p* < 0.0001) in birds that feed from the ground (69.1 %), being naturally exposed to tick attacks, than in arboreal bird species (13.6 %). The majority of ticks (*n* = 807; 96.7 %) were recovered from 56 ground-foraging birds of six species and only these hosts were included into further analysis. Forty-five (80.4 %) of the 56 infested birds carried simultaneously larvae and nymphs (Table 1). The highest intensity of infestation was found on the blackbird (21.1 ticks per infested bird; range 1–110) and the song thrush (13.1 ticks per infested bird; range 1–23) (excluding the single tree pipit infested by 36 ticks). These two bird species hosted 82 % (*n* = 681) of the ticks collected from all infested passerines.

### *Rickettsia* infection in bird-derived ticks

A total of 550 ticks from 53 birds were examined for the presence of *Rickettsia* spp. Of 295 larvae, 166 (56.3 %) were determined as unfed, 124 (42 %) as partially engorged, and five (1.7 %) as fully engorged. Among 255 nymphs, 62 (24.3 %) were unfed, whereas 188 (73.7 %) and five (2 %), respectively, were identified as partially and fully fed.

DNA of *Rickettsia* spp. were identified by the real-time PCR in 37.5 % (9 of 24) individually tested larvae and in 44.2 % of 95 larval pools. In the case of nymphs, 17.1 % (24 of 140) single specimens and 45.3 % of 53 pooled samples tested positive (Table 2). Rickettsia DNA was detected both in unfed larvae and nymphs (13.9 vs. 26.5 %), in partially engorged specimens (21.8 vs. 16.7 %), and in fully fed subadult ticks (10 %).

**Table 2:** Prevalence of *Rickettsia* spp. in immature *Ixodes ricinus* ticks feeding on ground-foraging passerine birds live-trapped in west-central Poland

Hosts with ticks		*Ixodes ricinus*				
		Larvae		Nymphs		Total
Species	n/n_1_	No. pools tested/no. ticks per 1 pool/no. positive pools (no. individual ticks/no/% positive) [no. of pools/no. /% positive]	No. ticks tested/minimal. no./(%) infected (MIR)	No. pools tested/no. ticks per 1 pool/no. positive pools (no. individual ticks/no./% positive) [no. of pools/no. (%) positive]	No. tested/min. no. infected/MIR	No. tested/min. no. infected/MIR
*Turdus merula*	26/19	10/1/3; 20/2/7; 25/3/7; 10/5/8	175/25/14.3	98/1/19; 28/2/12; 7/3/4	175/35/20.0	350/60/17.1
*Turdus philomelos*	11/8	4/1/3; 16/2/5; 5/3/4; 1/4/1	55/13/23.6	26/1/5; 7/2/3; 3/3/2	49/10/20.4	104/23/22.1
*Erithacus rubecula*	5/4	5/1/1; 5/2/4	15/5/33.3	5/1/0; 3/2/2	11/2/18.2	26/7/26.9
*Fringilla coelebs*	6/2	4/1/1; 4/2/0; 1/4/1	16/2/12.5	3/1/0	3/0	19/2/10.5
*Sturnus vulgaris*	5/2	1/1/1; 1/2/0	3/1	6/1/0; 3/2/1	12/1/8.3	15/2/13.3
*Anthus trivialis*	1/1	2/3/2; 5/5/3	31/5/16.1	2/1/0; 1/3/0	5/0	36/5/13.9
Total	53/35	(24/9/37.5); [95/42/44.2]	295/51/17.4	(140/24/17.1) [53/24/45.3]	255/48 /18.7	550/99/18.0

*n/n*_*1*_—no. infested birds tested/no. birds with infected ticks; *MIR* minimum infection rates (%)—at least one tick in each positive sample carried the pathogen

Combined data including both PCR-positive ticks tested individually and in pools showed that at least 17.4 and 18.7 % of larvae and nymphs, respectively, harbored rickettsiae (minimum infection pates (MIR): at least one tick in each positive sample carried the pathogen).

The highest MIR was noted for *E. rubecula* (27 %) followed by *T. philomelos* (22 %) and *T. merula* (17 %) (Table 2). PCR-positive ticks were found on each of the six passerine birds tested. Overall, 66 % of them carried at least one infected tick. The majority of PCR-positive tick samples (83.8 % of 99) tested individually and in pools derived from blackbirds and song thrushes.

### *Rickettsia* sp. identification

Seventy-one of 99 real-time PCR-positive tick samples produced amplicons in a regular and/or semi-nested PCR of partial regions of the *gltA* (*n* = 38) and 16S rRNA genes (*n* = 70). Sequence analysis of these amplicons revealed the presence of *R. helvetica* in 70 of them (Table 3). The 33 sequences obtained showed their 100 % homology with the corresponding fragments of *gltA*-gene of *R. helvetica* isolates obtained from *I. ricinus* in Poland (GenBank acc. no: EU779822; KJ740389), Slovakia (KF016135), Germany (KC0071266), and France (KF447530). Moreover, 68 sequences of the 16S rRNA gene fragments were 100 % identical to the corresponding sequence of *R. helvetica* clone CsFC (GQ413963) isolated from human cerebrospinal fluid in Sweden and to *R. helvetica* strain IR-698.9-AF (GenBank acc. no. KJ740388) from *I. ricinus* ticks derived from the yellow-necked mouse (*Apodemus flavicollis*) in Poland. The consensus sequence of 16S rRNA gene was deposited in GenBank database under acc. no. KU728665.

**Table 3:** Species identification of *Rickettsia* spp. in bird-feeding *Ixodes ricinus* ticks collected in the “Zielonka Forest” (west-central Poland) in 2006

*I. ricinus*		No. (%) of *Rickettsia*		
Stage	No. infected	*R. helvetica*	*R. monacensis*	Undetermined
Larvae	51	36 (70.6)	1 (2.0)	14 (27.4)
Nymphs	48	34 (70.8)	0	14 (29.1)
Total	99	70 (70.7)	1 (1.0)	28 (28.2)

*R. monacensis* (GenBank acc. no. KU728666) was identified only in one pool of five *I. ricinus* larvae from *T. merula* (Table 3). The obtained sequence was 100 % homologous to the sequences of corresponding fragments of *R. monacensis* strain IrR/Munich (GenBank acc. nos. LN794217 and NR_115686.1) derived from *I. ricinus*, Germany. It differed by one nucleotide from *Rickettsia* sp. IRS 3 and IRS4 (GenBank acc. nos. AF141907 and AF141908, respectively) from *I. ricinus*, Slovakia.

Twenty-eight *Rickettsia-*positive samples remain determined to the genus level due to the shortage of templates, the weakness of the some amplicons obtained, and too short or contaminated sequences received.

## Discussion

In this study, we investigated the frequency of *Rickettsia* spp. infection in bird-derived *I. ricinus* ticks which were collected from passerines mist-netted in two sylvatic habitats situated in the Landscape Park, Zielonka Forest, west-central Poland. In particular, a high prevalence of tick infestation was recorded in the group of ground-foraging and/or ground-nesting passerine species. Similar findings were reported throughout Europe (Michalik et al. 2008; James et al. 2013; Falchi et al. 2012; Hornok et al. 2014; Berthová et al. 2016). Birds feed large numbers of immature *I. ricinus* ticks. In our investigation, larvae clearly prevail over nymphs. It is in line with results obtained in Switzerland (Lommano et al. 2014) and Slovakia (Berthová et al. 2016) (62.2 vs. 37.8 % and 75.9 vs. 24.1 %, respectively). In other studies, however, almost the same proportion was noted (Falchi et al. 2012), or birds were found to be more heavily infested with nymphs than with larvae (Špitalská et al. 2006; Michalik et al. 2008; Franke et al. 2010a; Capligina et al. 2014).

Here, we report *Rickettsia* infection in *I. ricinus* ticks feeding on birds. Sequencing analysis of *gltA* and 16S rRNA genes confirmed that ticks were infected with at least two species of the spotted fever group: *R. helvetica* and *R. monacensis*. The sequences of the first species were identical or almost identical (>99 %) to published sequences of *R. helvetica* isolated from *I. ricinus* throughout Europe, including EU779822, KJ740389; KJ740388 from deer and rodents captured in the same area of investigation, and to the corresponding fragment of 16S rRNA gene from cerebrospinal fluid of Swedish patient with rickettsiosis. The sequence of the 16S rRNA gene of *R. monacensis* shared 99–100 % homology with *Rickettsia* sp. IRS 3 and IRS4, and with *R. monacensis* strain IrR/Munich derived from *I. ricinus* from Slovakia and Germany, respectively.

At least 12.7 % of investigated ticks were infected with *R. helvetica*. Field studies conducted in several European countries revealed different levels of *R. helvetica* infection in immature *I. ricinus* removed from passerines. Our results are similar to those reported from Switzerland (10.5 %) (Lommano et al. 2014), Russia (10.3 %) (Movila et al. 2011), and Latvia (12 %) (Capligina et al. 2014). The lowest infection rates were recorded in the Czech Republic (3 %) (Dubska et al. 2012) and Slovakia (5.9 %) (Berthová et al. 2016), whereas the highest value (∼53 %) in Hungary (Hornok et al. 2014). The prevalence of *R. monacensis* in analyzed ticks was significantly lower—0.2 %. This finding corroborates results obtained in studies conducted in Switzerland, Slovakia, and Spain where 0.4–0.5 % *I. ricinus* from migratory birds were found to be infected with *R. monacensis* (Palomar et al. 2012; Lommano et al. 2014; Berthová et al. 2016). In contrast, in Germany, Russia, Hungary, and Sweden recorded infection rates were much higher—3.1, 3.9, 7.9, and 8.3 %, respectively (Hildebrandt et al. 2010; Movila et al. 2011, Elfving et al. 2010; Hornok et al. 2014). All reports, however, emphasize the role of birds in the natural cycle of tick-borne pathogens.

It is worth mentioning that throughout Europe, numerous studies revealed great variability of prevalence of *Rickettsia* spp. also in questing *I. ricinus*, ranging from 1.5 % in Finland (Sormunen et al. 2016) up to an exceptionally high infection rate in a vegetation-rich dune area in The Netherland ∼66 % (Sprong et al. 2009). These differences may reflect variations not only in spatial but also in seasonal dynamics of the pathogen and ticks themselves. For instance, in Hamburg, Germany, significantly lower prevalence of *R. helvetica* was observed in ticks collected in spring: 36.5–29.5 % compared to summer and fall: 55.0–64.5 % (May and Strube 2014). Thus, it should be taken into consideration that results of particular studies provide information on the current situation in a given place and time.

*Rickettsia* spp. was detected in ticks which fed upon 66 % of infested birds. Infection was confirmed in unfed, partially, and fully fed larvae and nymphs. The presence of rickettsiae in the immature ticks may result from acquisition bacteria through a transovarial route or through a blood meal from a rickettsiemic bird. Bacteria could have been also transmitted between co-feeding ticks which are often aggregated around the beak or the eyes of infested birds (Elfving et al. 2010). All ticks analyzed by us derived from birds which had been previously proved to be non-rickettsiemic (Stańczak et al. 2009). Moreover, all *Rickettsia-*positive specimens occurred simultaneously with non-infected ticks on the same bird hosts, being often attached in close proximity to each other. For instance, among 66 ticks unfed or in different stages of engorgement, found on a single blackbird, only nine were considered as infected (MIR). Thus, we suppose that PCR-positive ticks did not acquire rickettsiae via a blood meal. However, we cannot exclude that the bacterial load in host blood samples tested by us was too low to be detected by a conventional PCR. Hornok et al. (2014) using TaqMan real-time PCR method have reported for the first time *R. helvetica* bacteremia in six (4.7 %) of 128 blood samples obtained from birds sampled in Hungary. The bacterium was detected in five robins and a single dunnock (*Prunella modularis*). Recently, an active rickettsiemia has also been detected in 4.2 % blood samples of nine different bird species from Slovakia (Berthová et al. 2016). In both studies, the range of Ct values (33–40) reflected low-to-medium levels of bacterial loads. The authors suggested that rickettsiemia may last after detachment of the vector tick in relevant avian hosts, and rickettsiemic birds may provide a source of infection for *I. ricinus*, but efficacy of transmission is low.

There is no data on the prevalence of *Rickettsia* infection in questing subadult *I. ricinus* in the investigated area; however, we could compare infection rates in ticks feeding on different hosts. The minimum infection rates (MIR) of larvae (17.4 %) and nymphs (18.7 %) collected from birds were 1.6 and 2.3 times higher, respectively, than the MIR of larvae (10.7 %) and nymphs (8.3 %) detached from rodents (*A. flavicollis* mice and *Myodes glareolus* voles) trapped there in 2006 (Biernat et al. 2016). Bird-feeding immature *I. ricinus* were at least twice more frequently infected with *Rickettsia* spp. than nymphs feeding on deer (8.4 %) and showed a comparable infection rate with female ticks collected from these hosts (16.8 %) (Stańczak et al. 2009). It is worth mentioning that in questing *I. ricinus*, the prevalence of infection in females usually exceeded infection in nymphs (Stańczak et al. 2008, Reye et al. 2010; Silaghi et al. 2011). Furthermore, the prevalence of *R. helvetica* in feeding ticks collected from different vertebrate hosts (birds, rodents, and cervids) in the Landscape Park, Zielonka Forest, was higher in comparison with the infection rates detected in questing nymphs (4.9 %), male (5 %), and female (7 %) ticks sampled in several forest habitats in the Wielkopolska province (Stańczak et al. 2008). These data may indirectly support suggestion that in sylvatic habitats in Wielkopolska province of western Poland, passerine birds seem to play a key role in the ecology of SFG *Rickettsia* as the most probable natural hosts for these bacteria and carriers of infected tick vectors.

Except for *Anthus trivialis*, the remaining bird species investigated in our study which carried infected ticks are frequently noted also in periurban or urban habitats. Thus, the obtained data indicate a risk for the import of tick-borne rickettsiae by birds to the human vicinity. For instance, the overall prevalence range of rickettsiae, including *R. helvetica* and *R. monacensis*, in ticks collected from synanthropic birds in Hungary was 29–40 % (Hornok et al. 2013). In this country, both species have been recently noted in questing *I. ricinus* in urban parks with the 44.6 % prevalence of *R. helvetica* in adult females (Szekeres et al. 2016). Earlier, they were recorded in city parks in the Czech Republic (2.2 %), Slovakia (8.3–14.5 %), France (5.8 %), Poland (3.7–5.9 %), and Germany (1–30.4 %) (Rizzoli et al. 2014) with highest overall infection rate (52.5 %) reported in the city of Hamburg (May and Strube 2014). Both *R. helvetica* and *R. monacensis* are proved to be etiologic agents of human rickettsioses. Human cases, including an acute febrile illness, meningitis, and a fatal perimyocarditis, caused by *R. helvetica* have been reported so far mostly from Sweden (Nilsson 2009; Nilsson et al. 1999, 2010, 2011) and France (Fournier et al. 2000). *R. monacensis* infection has been diagnosed in two patients in Spain (Jado et al. 2007) and in one patient in Italy (Madeddu et al. 2012) with Mediterranean spotted fever-like illness.

The results of the current study confirm and strengthen our previous findings that in the sylvatic habitat in Wielkopolska province, infection of *I. ricinus* with SF *Rickettsia* spp., predominantly *R. helvetica*, is frequent. Hard ticks can transmit them transstadially and transovarially and serve both as vectors and reservoirs of these pathogens (Rizzoli et al. 2014). Rodents, deer, birds, on which ticks abundantly feed, are naturally exposed to rickettsiae via infected ticks but their ability to transmit the pathogen to naive feeding specimens seems to be limited, due to the short-lasting rickettsiemia or possible low load of bacteria. However, their role in the maintenance of rickettsiae in nature by spreading them to non-infected ticks horizontally should not be excluded. Especially, reservoir competence of birds should be taken into consideration.
